# Conversation with Chen-Ning Yang: reminiscence and reflection

**DOI:** 10.1093/nsr/nwz113

**Published:** 2019-08-07

**Authors:** Mu-ming Poo, Alexander Wu Chao

**Affiliations:** 1 Center for Excellence, Brain Science and Intelligence, Chinese Academy of Sciences; 2 Stanford University; 3 Executive Editor-in-Chief of NSR

## Abstract

*Chen-Ning Yang (* 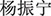 *) is the most distinguished Chinese theoretical physicist. In 1954, together with Robert Mills, he formulated the Yang–Mills Gauge Theory, which led to the development of the Standard Model, the leading framework for understanding particle physics. In 1956, Yang and Tsung-Dao Lee (* 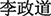 *) proposed the possibility of parity non-conservation in weak interaction, which won them the Nobel Prize in Physics in 1957. Besides these two major achievements, Yang made many other seminal contributions to particle physics, statistical physics and condensed matter physics.*

*At the end of 2003, Yang returned to China from the US and established the Institute for Advanced Study at Tsinghua University in Beijing. *NSR*’s Executive Editor-in-Chief Mu-ming Poo (* 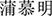 *), a neurobiologist, and Alexander Wu Chao (* 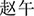 *), an accelerator physicist at Stanford University, talked with Professor Yang on a variety of topics, ranging from his retrospective view on Yang–Mills theory, on his contemporary physicists, on tastes in scientific research, and on the current and future developments of Chinese science. The following is an excerpt from this conversation that took place on 21 March 2019 at Tsinghua University, Beijing.*

## Yang–Mills gauge theory

**Chao:** Let’s start with Yang–Mills theory. Among the many important contributions you made, people consider Yang–Mills theory to be the most important. Do you agree? Why do you think it is so important?

**Yang:** In the field of basic theoretical physics, it is generally believed that Yang–Mills gauge theory is one important element at its foundation. When it was first published in the 1950s, most people did not expect it to survive. However, by the 1970s, after the first round of experimental evidences, and after some important additional insights were introduced, namely the idea of symmetry breaking, it gradually evolved into the Standard Model, which has become the most important development in fundamental physics in the second half of the 20th century.

How about the future? In a 1980 article I wrote about Einstein, I coined the phrase, ‘Symmetry dictates interaction’. It means that the structure of all forces in the universe are intimately related to the concept of symmetry. In Chinese it becomes 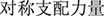. I believe this short phrase captures the spirit of why gauge theory is so important. It also captures the direction of developments in theoretical physics in the future.

Among the questions we have not yet answered, the most important are how to integrate gravity into the system, and how to unify the system. I believe, and most physicists believe, that the way to accomplish these is to introduce more symmetry. Searching for these additional symmetries has been the concentrated effort in the past 40 years. But so far it has not succeeded.

**Chao:** This new symmetry surely is not a simple symmetry like left–right symmetry?

**Yang:** Yes. Certainly it will be more subtle, more intricate.

The concept of symmetry exists, for example, already in early Chinese culture, through couplets (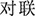), which is one simple but important manifestation of symmetry in Chinese literature.

 

In Western culture, the Greeks already very much emphasized symmetry. They believed that the principles of symmetry govern everything in the world, which may be considered the ancient philosophical version of ‘symmetry dictates interaction’. In contrast, the modern version needs to be formulated in precise mathematical language.

**Figure 1:**
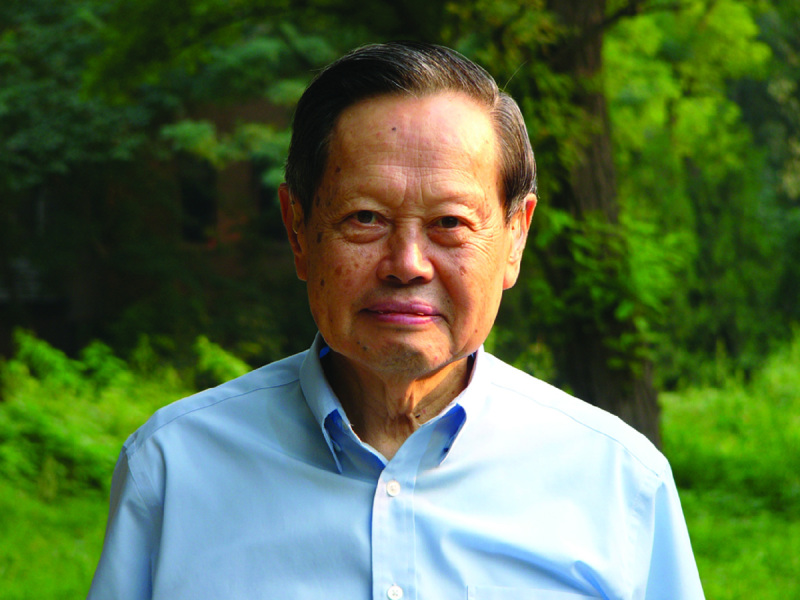
Theoretical physicist, Professor Chen-Ning Yang *(Courtesy of Professor Chen-Ning Yang*).

The name gauge theory is not my invention. Around the mid-1910s Einstein said that now we have two field theories: one, electromagnetism, and the other, gravity. We should try to unify them, formulating a unified theory. Einstein himself continued to work over the next few decades on this unification idea, but did not succeed. Then in 1918 Hermann Weyl took up the challenge and found a new symmetry in Maxwell’s electromagnetic equations. This new symmetry was related to the idea of parallel displacement in general relativity, and was tied to changing scales, i.e. to changing gauges. Thus the name gauge theory was coined by Weyl.

**Poo:** You listened to Einstein’s lectures in 1949, and you developed the Yang–Mills theory in 1954. Are there connections between them, or were you influenced by him?

**Yang:** I was already influenced by Einstein’s call for unification of different forces of nature, and by Weyl’s gauge symmetry formulation of electromagnetism, when I was a graduate student in Chicago.

But a more immediate drive for me was that in the decade after 1945 the most exciting development in physics was the many experimentally discovered new elementary particles. How do they interact with the old particles, with protons, neutrons, electrons, and among themselves? There were many papers written, theoretical and experimental, about how this θ particle interacts with that K particle, how Σ and Λ particles interact, etc. As a graduate student in the University of Chicago, I thought a general principle of interactions was needed, and that principle may come from Weyl’s gauge symmetry.

It happened at that time that an important topic in particle physics was isotopic spin symmetry, an SU2 symmetry first discovered in the 1930s in nuclear physics. The new particles were naturally classified according to their isotopic spin values: 1 or 1/2 or 3/2, etc. I have always liked symmetry considerations and Group Theory. Thus it occurred to me that one should generalize Weyl’s gauge symmetry from a U1 symmetry to an SU2 symmetry.

**Poo:** So it means to start with mathematical insight to order physical reality. We all say that this is a unique approach of yours.

**Yang:** But it was not all smooth sailing because U1 symmetry is a commuting symmetry and SU2 symmetry is a non-commuting symmetry: while the first steps to a non-commuting theory were mathematically easy, the next steps led to formulae that became more and more complicated, and I had to give up. Then, in the next few years, more new particles were discovered, and the need to formulate a general principle of interactions became more urgent for me, and I naturally returned to the attempt again, only to reach the same dead end.

Between 1947 and 1954 I must have repeated this unsuccessful attempt three or four times. Then came 1953–54 when I visited Brookhaven National Laboratory, where I shared an office with Mills, a very bright young fresh PhD, and a devout Christian, I should add. We naturally discussed many topics in physics, including my failed attempts at creating an SU2 gauge theory. During one of these discussions, we observed that the undesired complicated terms were quadratic and cubic. Could they be cancelled if we introduce quadratic and/or cubic terms at the beginning? It turns out that a simple quadratic term introduced at the beginning did miraculously cancel all the undesired complicated terms! The cancellation was so beautiful we knew we had hit a gold mine.

**Figure 2:**
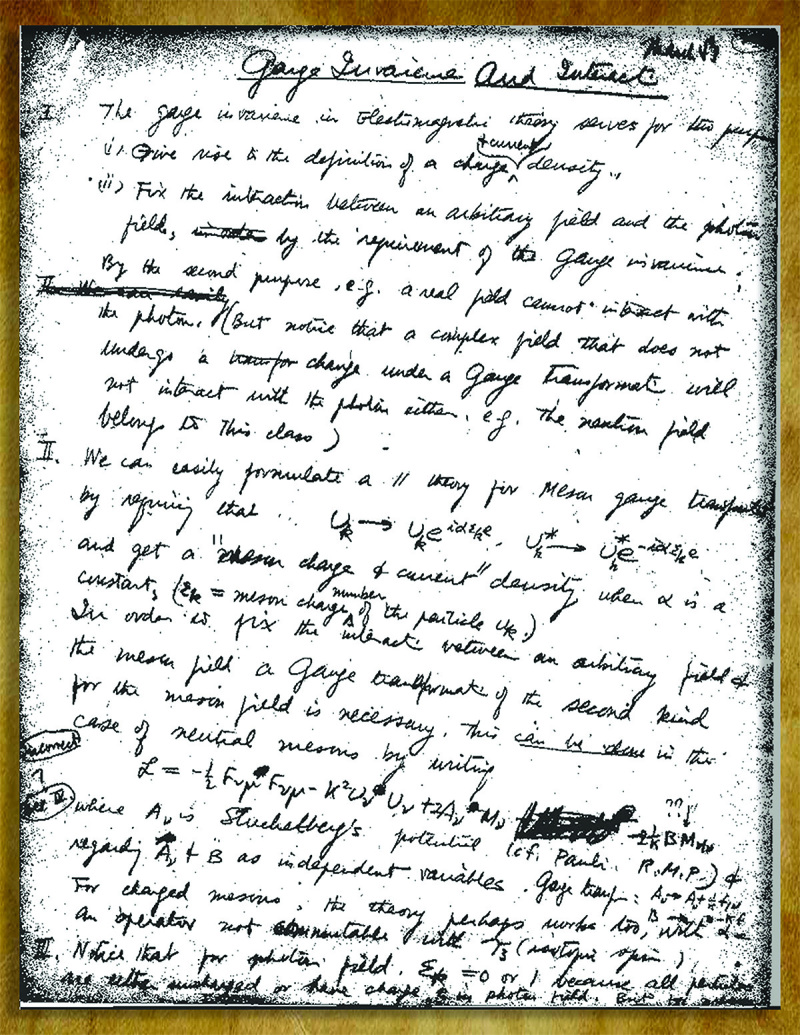
A page from C.N.Y.’s 1947 notes *(Courtesy of Professor Chen-Ning Yang)*.

**Chao:** It was all smooth sailing after that?

**Yang:** No. The theory seemed to require the existence of charged massless particles, which for many reasons cannot be!

In February 1954 Robert Oppenheimer, the then President of the Princeton Institute for Advanced Study, heard about our work and invited me to return to Princeton to give a lecture about our theory. I did, and Wolfgang Pauli was in the audience.

He had done similar work and had encountered the same problem of massless charged particles. He gave me a hard time at the lecture which I had later described in detail in my *Selected Papers with Commentary.* Back in Brookhaven I worked hard for several months with Mills to try to overcome the difficulty, without success.

The theory was very beautiful. Should it be published even though it has an unresolved difficulty?

We agonized over this question and finally decided it should be published, because it was very very beautiful. Furthermore, field theory was not mathematically defined. It was beset with divergence difficulties, with the mysterious success of the renormalization program. Thus our difficulty may somehow be resolved in future developments in field theory.

Pauli chose not to publish.

**Poo:** Thanks for this detailed description of the birth of Yang–Mills theory. We have a question for you about what came after Yang–Mills. How was the massless charged particle difficulty resolved later?

**Yang:** It was resolved through adding a new idea called symmetry breaking. With this idea a quantitative understanding of three forces of nature (i.e. strong forces, electromagnetic forces and weak forces) was beautifully made, resulting in what is now called the Standard Model. And this model has been successfully tested experimentally in hundreds of experiments. It should be regarded as the triumphant grand contribution of the experimental and theoretical high energy physics community in the last 60 or 70 years. It had won several Nobel Prizes for about a dozen theoretical and experimental physicists.

## Fiber bundle theory, math and physics

**Poo:** You have been credited with bringing math and physics together in the 1970s. Please tell us the story.

**Yang:** In 1975 Tai Tsun Wu (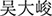) and I published a paper about the physicist’s electromagnetic field theory and its relationship with the mathematician’s fiber bundle theory. To clarify the deep precise relation between these two theories we constructed a dictionary. It happens that in 1976 Isadore Singer visited Stony Brook and I gave him a copy of our reprint. He took it to Oxford to show it to Michael Atiyah and other mathematicians; they were deeply interested and began to work on gauge fields and related topics, leading to a period of close collaboration between mathematicians and physicists. I believe this is an important event in the long history of both disciplines. In 1988 Singer, in an article about Weyl, recounted this story. Reprinted below is the Wu–Yang dictionary in Singer’s article.

**Figure 3:**
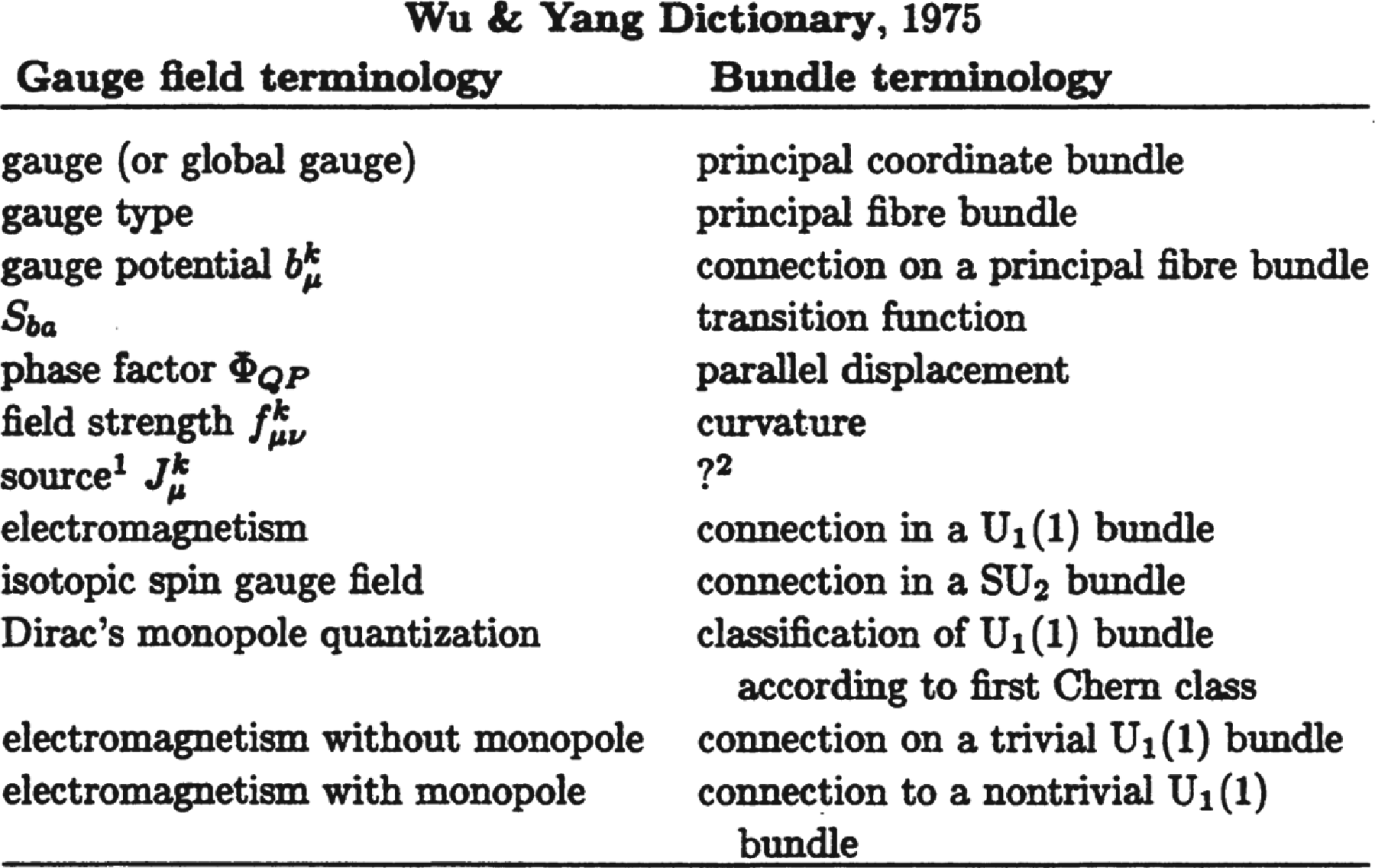
The Wu-Yang dictionary in Singer's 1988 article. Reprinted with the permission of the American Mathematical Society.

For me, the recognition that gauge theory equations are SPECIAL CASES of equations in a subtle field of mathematics called fiber bundles was at once exhilarating and humbling. In a 1979 article celebrating the 100th anniversary of the birth of Einstein, I had recounted a conversation with Shiing-Shen Chern (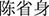), one of the founding mathematicians of fiber bundle theory:

In 1975, impressed with the fact that gauge fields are connections on fiber bundles, I drove to the house of Shiing-Shen Chern in El Cerrito, near Berkeley. (I had taken courses with him in the early 1940s when he was a young professor and I an undergraduate student at the National Southwest Associated University in Kunming, China. That was before fiber bundles had become important in differential geometry and before Chern had made history with his contributions to the generalized Gauss–Bonnet theorem and the Chern classes.) We had much to talk about: friends, relatives, China. When our conversation turned to fiber bundles, I told him that I had finally learned from Jim Simons the beauty of fiber-bundle theory and the profound Chern–Weil theorem. I said I found it amazing that gauge fields are exactly connections on fiber bundles, which the mathematicians developed without reference to the physical world. I added ‘this is both thrilling and puzzling, since you mathematicians dreamed up these concepts out of nowhere.’ He immediately protested, ‘No, no. These concepts were not dreamed up. They were natural and real.’

## On tastes in doing science

**Poo:** Freeman Dyson once called you a conservative revolutionary. Do you agree?

**Yang:** Before he coined that phrase, I had never thought I was conservative. After his speech in 1999 with that title I thought about it and felt that he was quite right: I value tradition and tend to strike out in new directions only when necessary.

**Poo:** This is related to taste in doing research. You have often said that in doing science it is important to have one’s own taste.

**Yang:** Yes, and I would add: it is important not only for issues with large impact. Even for a graduate student it is important to develop one’s own taste: what ideas, what types of questions, what types of approach to concentrate on, etc. Taste formation is influenced by many factors: native talent, family environment, early teachers, one’s own temperament and luck.

**Poo:** How did you develop your taste in physics?

**Yang:** I was gifted in math, but was discouraged to go into math by my father who thought math was not what China needed. I was extraordinarily lucky in college, in wartime Kunming, to have two professors, Ta-You Wu (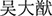) and Jwu-Shi Wang (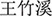), who channeled me into fields of physics that later developed into major areas of research. Working in these two nascent fields greatly influenced my taste and style in research.

## On development of science in China

**Yang:** You (Poo) have said that Chinese scientists are particularly hard-working, dedicated and persistent. That is the reason why the complete artificial synthesis of bovine insulin and the development of the anti-malaria drug artemisinin were all striking early achievements in China when the country was still very backward industrially.

I agree, and would like to add some additional points.

China has her special traditional Confucian culture, which values hard work and patience, puts family and country before the individual and greatly values education.

**Poo:** I agree, of course. But is the Confucian tradition too conservative?

**Yang:** I agree that compared to American tradition Chinese tradition is conservative. But is that bad? My answer: bad in some respects. But I would fault the American tradition more. It is too ‘progressive’.

**Chao:** Is the rapid economic growth in China connected to the culture issue?

**Yang:** I believe very closely connected.

**Chao:** So from the point of view of economic growth, is it that being conservative and conforming to authority are good things? Everybody should work toward the same goal?

**Poo:** Fine. But being too conservative may slow scientific development.

**Yang:** Yes. To decide for a nation whether to be conservative or progressive in scientific development is a highly complex problem.

I believe in two basic principles. One, national interest always over group interest. Two, adopt the middle philosophy (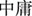): not going to extremes.

**Poo:** At present I feel there is much wrong information and misinterpretation about the developmental policies of China. For example the Belt and Road Initiative is a cooperative and friendly project. But in American eyes China is trying to seek hegemony.

**Yang:** America is afraid of Chinese success, not only in the Belt and Road Initiative, but also in the Chinese projects in Africa. These successes I believe are deeply related to the Confucian attitude about human relations, which is very very different from that of the Americans, the British and the Japanese. I don’t know whether anyone has done research on this important topic.

**Poo:** The world is now inflicted by populism, extreme nationalism and racism. Global scientific and technological development will inevitably be influenced by these. What is your view on this?

**Yang:** I agree.

**Chao:** The westerners had treated Africa as colonies. The purpose of being there was to exploit the resources. The Belt and Road is different; basically it is a mutually beneficial win–win scheme.

**Yang:** From a bigger picture, Chinese tradition has been talking about ‘education without categorization’ (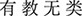) since Confucius’ time. Therefore phenomena such as the caste system in India did not take place in China. The primitive concept of ‘not my kind’ is biological. It is overcome in the Chinese social system through education, through education from early babyhood, emphasizing the importance of harmony (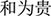).

## On initiating international big science projects

**Poo:** Perhaps influenced by political and economic competition, there have been talks by top leadership pronouncing that China should initiate international big science projects based in China, rather than just being a minor participant in such projects as in the past. Proposals for such projects are being formulated. This appears to be a government mandate for Chinese scientists in the near future. Do you think this is realistic expectation?

**Yang:** In those areas where Chinese scientists have become world leaders, I think to establish such large projects is worthwhile. But it will consume a lot of resources.

**Poo:** There is now consensus in and out of the government that China needs to raise its support to basic research, which currently amounts to only 0.7% of GDP, much lower than that of the USA and the European Union. The key question is: in which way will basic research be supported, is it for big science or small science? You can put lots of resources into constructing a big research facility for basic research, or you can greatly increase the support of many small laboratories and individual investigator-initiated projects.

**Yang:** This is my opinion: the development of the Chinese economy in the past 40 years is a shining success. And forward-looking large projects have played essential roles in this success. But that model does not work for the development of basic science because revolutions in basic science always originate from a few individuals’ efforts, never from large projects. Electromagnetism, Darwin’s theory, fission, the semiconductor, the double helix, penicillin, all of these great revolutions in basic sciences have come from research by a few individuals with a small budget, never from big projects.

## RefLections, regrets and advice

**Chao:** You have made significant contributions to science and society over your career. You have made many decisions along the way. In retrospect, do you have any regrets? Perhaps different decisions should have been made?

**Yang:** Yes, of course I have regrets. Let me begin with my research in physics. The big mistake was my dismissal of ‘symmetry breaking’ in the 1960s. I have discussed this in my *Selected Papers with Commentary.*

Other than that, I have no important regrets in my research in physics. I made many correct and important personal decisions in my long career: I visited China in 1971, and later in around 2003 decided to return completely to China. In 2004 I married Fan Weng (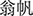), who was 54 years younger than I. These were all right decisions.

**Chao:** Finally, we have a simple question. We would like to know if you have any advice to the young people? Many young people work very hard, but they do not know how they can do better.

**Yang:** I think Chinese young students often ignore the importance of their own interests, which maybe a result of China’s special cultural and educational system. They are taught to conform to the needs of society, but not to explore and fulfill their own interests. So I suggest that Chinese young students pay more attention to the development of their own interests. Meanwhile, if you ask me to give suggestions to American students, I would recommend that they pay less attention to some of their so-called interests and consider more the major developmental trends of society and science.

Of course, there is also a suggestion for Chinese parents and teachers: please encourage and foster the interests of the young.

## Notes

Mu-ming Poo is the Executive Editor-in-Chief of NSR and the Scientific Director of the Center for Excellence in Brain Science and Intelligence, Chinese Academy of Sciences; Alexander Wu Chao is an accelerator physicist and a Professor at Stanford University.

